# Gene silencing of *NOB1* by lentivirus suppresses growth and migration of human osteosarcoma cells

**DOI:** 10.3892/mmr.2014.2119

**Published:** 2014-04-04

**Authors:** BINGPENG CHEN, JINGJING LIU, DANKAI WU, YANGUO QIN, CHUANGANG PENG, CHEN LI, JINCHENG WANG

**Affiliations:** 1 Department of Orthopedics, The Second Hospital of Jilin University, Changchun, Jilin 130041, P.R. China; 2 Department of Oncology, Jilin Tumor Hospital, Changchun, Jilin 130021, P.R. China

**Keywords:** NIN1, RPN12 binding protein 1 homolog *(S. cerevisiae)*, short hairpin RNA, osteosarcoma, migration

## Abstract

NIN1/RPN12 binding protein 1 homolog (*Saccharomyces cerevisiae*) (*NOB1*) encodes a chaperone protein that joins the 20S proteasome with the 19S regulatory particle in the nucleus and facilitates the biogenesis of the 26S proteasome, which plays a role in maintaining cellular homeostasis by controlling protein degradation. In order to investigate the role of *NOB1* in osteosarcoma, *NOB1* protein expression in human osteosarcoma cell lines was assessed using western blot analysis. Lentivirus-mediated short hairpin RNA was employed to knock down *NOB1*, and the effects of *NOB1* silencing on cell growth were assessed using MTT, colony formation and cell cycle assays. Cell migration was observed using the Transwell assay. In addition, the expression levels of E-cadherin and β-catenin were examined by western blot analysis. Functional analysis indicated that *NOB1*-knockdown markedly inhibited cell growth and caused G2/M-phase arrest in human osteosarcoma cells. Furthermore, *NOB1* inhibition decreased cell migration and increased E-cadherin and β-catenin expression in U2OS cells. In conclusion, the present study suggested that *NOB1* depletion may inhibit osteosarcoma development by increasing E-cadherin and β-catenin expression and, for the first time, indicated the potential of *NOB1* as a target in osteosarcoma treatment.

## Introduction

In eukaryotic cells, the process of proteolysis is executed by a diverse group of enzymes known as proteases, and the ubiquitin-proteasome pathway (UPP) is the most significant intracellular proteolytic pathway. The degradation process mediated by the UPP involves two steps: i) The target proteins are ubiquitinated by multiple ubiquitin molecules; and ii) the tagged proteins are recognized and degraded by the major ATP-dependent protease, the 26S proteasome complex (1). The 26S proteasome is a biological macromolecule containing a 20S catalytic core and two 19S regulatory complexes (2). Selective degradation of proteins by the UPP is a critical determinant for maintaining cellular homeostasis. Numerous proteasome target proteins are involved in the regulation of cancer cell proliferation, differentiation and apoptosis (3,4). Therefore, the aberrant degradation of oncoproteins or tumor suppressor proteins can result in uncontrolled cell growth in numerous cancer types.

*In vitro* and *in vivo* studies have demonstrated that inhibition of proteasomes is an effective anticancer therapeutic approach. Bortezomib, one of the first proteasome inhibitors, which was designed to inhibit the activity of the 26S proteasome by binding to the N-terminal threonine residues at the active site of the catalytic region (5), was shown to be efficient against a variety of malignancies, including myeloma, chronic lymphocytic leukemia and certain solid tumors (6–8). Osteosarcoma is the most common primary bone sarcoma and mostly affects adolescent patients. Since osteosarcoma is metastatic and highly aggressive, novel treatment strategies must be developed. Bortezomib has been shown to suppress growth and induces apoptosis in osteosarcoma cells and xenografts (8). The thiazole antibiotic thiostrepton, which has been identified as a proteasome inhibitor in mammalian tumor cells (9), induces apoptosis in a wide variety of human cancer cell lines, including osteosarcoma cells, on its own or in combination with bortezomib (5,10). These data strongly suggest that proteasome inhibition may also be effective as an adjuvant to current treatment regimens for osteosarcoma.

NIN1/RPN12 binding protein 1 homolog (*NOB1*), which was firstly identified in *Saccharomyces cerevisiae*, encodes the essential protein Nin one binding protein (NOB1p) (11). As a chaperone protein, NOB1p joins the 20S proteasome with the 19S regulatory particle in the nucleus and facilitates the maturation of the 20S proteasome, thereby favoring the completion of 26S proteasome biogenesis (12). Furthermore, *NOB1*, along with five other genes, has been used as a diagnostic marker discriminating chronic phase from blast crisis chronic myelogenous leukemia (13). RNA interference (RNAi)-mediated downregulation of *NOB1* suppresses the growth of human ovarian cancer cells and hepatocellular carcinoma cells (14,15). In the present study, short hairpin RNA (shRNA) was employed to knock down *NOB1* in osteosarcoma cells, and the effects of *NOB1* silencing on cell growth and migration were explored.

## Materials and methods

### Materials

SF-86, Saos-2, MG63, SW1353 and U2OS human osteosarcoma cells and HEK-293T cells were obtained from the Type Culture Collection of the Chinese Academy of Sciences (Shanghai, China). TRIzol^®^ reagent was purchased from Invitrogen Life Technologies (Carlsbad, CA, USA). The SYBR^®^ Green Real-Time PCR assay kit was obtained from Applied Biosystems, Inc. (Beijing, China). Rabbit anti-NOB1p antibody was purchased from Sigma-Aldrich (St. Louis, MO, USA). Anti-GAPDH antibody and goat anti-mouse immunoglobulin G conjugated with horseradish peroxidase antibody were obtained from Santa Cruz Biotechnology, Inc. (Santa Cruz, CA, USA). Enhanced chemiluminescence reagents were purchased from Amersham Life Science (Arlington Heights, IL, USA).

### Cell culture

Cells were grown in Dulbecco’s Modified Eagle’s medium (DMEM; HyClone, Logan, UT, USA) containing 10% fetal bovine serum (FBS; Invitrogen Life Technologies) at 37°C under 5% CO_2_.

### Lentivirus production and lentiviral transduction

shRNA targeting the *NOB1* (CCGGGCTGAACAATTTCAGTCATT TCTCGAGAAATGACTGAAATTGTTCAGCTTTTTG) and negative control (TTCTCCGAACGTGTCACGT) sequences were cloned into pFH-L (Shanghai Hollybio, Shanghai, China). The reconstructed pFH-L-sh*NOB1* and pFH-L-shCon vectors were then co-transfected into HEK-293T cells together with the helper plasmids pVSVG-I and pCMVΔR8.92 (Shanghai Hollybio) to generate lentiviruses. After 96 h of incubation, the lentiviral particles were harvested from the supernatant by ultracentrifugation (16,17). The RNAi lentiviruses were referred to as sh*NOB1* for the specific interference with the *NOB1* gene and shCon for the negative control. For lentiviral transduction, 40% confluent Saos-2/U2OS osteosarcoma cells were incubated with Lv-sh*NOB1* or Lv-shCon for 96 h, with a replacement of the media 24 h after lentiviral treatment.

### Quantitative polymerase chain reaction (qPCR)

Saos-2 and U2OS cells were cultured in six-well plates and then infected with the sh*NOB1* or shCon lentiviruses. After 96 h of incubation, total RNA was isolated from cultured cells using TRIzol^®^ reagent and then cDNA was synthesized from total RNA. Two sets of primers were used for PCR: β-actin (*ACTB*) forward, 5′-GTGGACATCCGCAAAGAC-3′ and reverse, 5′-AAAGGGTGTAACGCAACTA-3′; *NOB1* forward, 5′-GAAAGAACAACGCCCTGGAG-3′ and reverse, 5′-CAGCCTTGAGATGACCTAAGC-3′. qPCR was performed according to the manufacturer’s instructions (Applied Biosystems, Inc.). The relative mRNA expression of *NOB1* was calculated using the 2^−ΔΔCt^ method (18).

### Western blot analysis

Whole cell extracts were prepared with ice-cold cell lysis buffer (10 mM Tris-HCl, pH 7.4, 1 mM EDTA, 0.1% Triton X-100 and 0.1% SDS) and the protein concentration was determined using a Bradford assay kit (Pierce Biotechnology, Inc., Rockford, IL, USA). Protein extracts were separated using SDS-PAGE, transferred onto a nitrocellulose membrane and incubated with anti-NOB1p and anti-GAPDH antibodies. Immunodetection was performed using an enhanced chemiluminescence western blotting kit (Amersham Biosciences Inc., Piscataway, NJ, USA).

### Cell proliferation assay

Cells collected from the three groups (Lv-sh*NOB1*, Lv-shCon and control) were trypsinized, resuspended, seeded into 96-well plates at a density of 2,000 cells per well and then incubated at 37°C for 96 h after lentiviral treatment. The number of viable cells was assessed at indicated time-points, when 20 μl MTT solution (5 mg/ml) was added into each well. The plate was incubated for 4 h. The fixed plate was then washed and 100 μl dimethyl sulfoxide was added. The absorbance of each plate was measured at 595 nm using a spectrophotometer.

### Colony formation assay

Following infection, cells in the three groups (Lv-sh*NOB1*, Lv-shCon and control) were seeded into a six-well plate at a density of 300 cells per well and maintained at 37°C for 13 or 14 days (U2OS2 and Saos-2 cell lines, respectively). The culture media were changed every 2–3 days. When the colonies were formed, the plate was washed and fixed, stained with Giemsa (Sigma Chemical Co.) for 10 min, and washed three times with double-distilled H_2_O. The stained cells and colonies (>50 cells/colony) were photographed and counted.

### Cell cycle analysis

The cell cycle distribution (G0/G1, S or G2/M phase) was characterized by differences in DNA content via flow cytometry. Cells were collected by centrifugation at 404 × g for 5 min, washed with phosphate-buffered saline (PBS) and fixed in ethanol. The fixed cells were then resuspended in propidium iodide/RNase/PBS for incubation in the dark (37°C, 30 min). The stained cells were analyzed using the FACSCalibur™ II sorter and the CellQuest™ fluorescence-activated cell sorting system (BD Biosciences, San Diego, CA, USA). The percentage of cells in each cell cycle phase was analyzed. Each experiment was repeated three times and the results are shown as the average.

### Cell migration assay

The motility and migration of U2OS cells were evaluated using the Transwell assay. Briefly, trypsinized U2OS cells were transferred into the upper chambers of the Transwell plates (8.0-μm pores, Corning Costar, Cambridge, MA, USA). The lower chamber was filled with 500 ml DMEM supplemented with 10% FBS. After 24 h at 37°C under 5% CO_2_/95% air, cell migration was determined by counting cells in the bottom of the membrane stained with crystal violet, and scored visually in five random fields using light microscopy (magnification, ×100). In addition, migrating cells were dissolved and quantified at 570 nm using a spectrophotometer.

### Statistical analysis

Data are expressed as the mean ± standard deviation of at least three independent experiments. Statistical significance was assessed using the Student’s t-test. P<0.05 was considered to indicate a statistically significant difference.

## Results

### Knockdown of NOB1 by the shRNA lentivirus system in osteosarcoma cells

**Figure 1:**
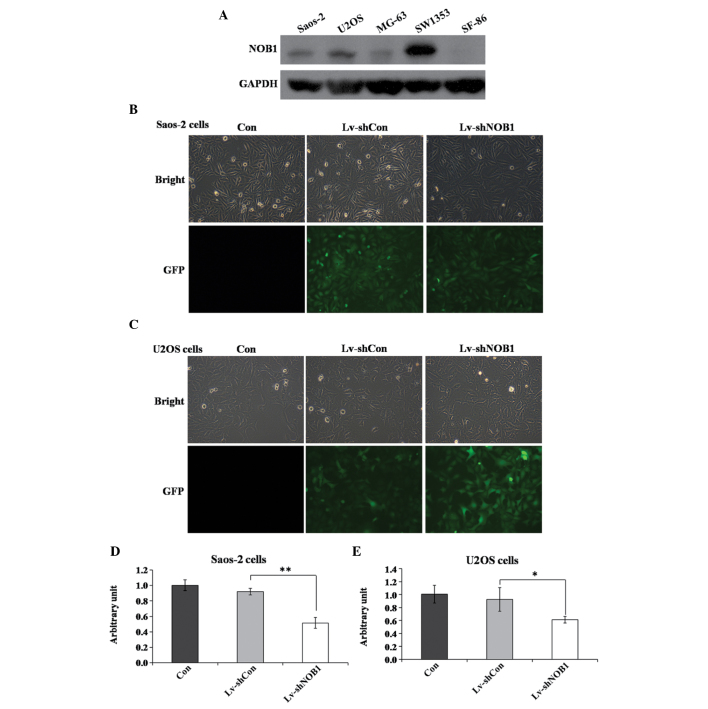
*NOB1*-knockdown by a lentivirus-mediated RNA interference system. (A) Expression levels of Nin one binding protein in five osteosarcoma cell lines (Saos-2, U2OS, MG-63, SW1353 and SF-86) using western blot analysis. (B and C) Fluorescence photomicrographs of (B) Saos-2 and (C) U2OS cells infected by the lentivirus. Pictures were captured 96 h after infection (magnification, ×100). (D and E) Identification of *NOB1*-knockdown efficiency via quantitative polymerase chain reaction in (D) Saos-2 and (E) U2OS cells. ^*^P<0.05, ^**^P<0.01 compared with Lv-shCon. Con, no lentivirus treatment; Lv-shCon, control lentivirus; Lv-sh*NOB1*, lentivirus containing short hairpin RNA targeting *NOB1; NOB1*, NIN1/RPN12 binding protein 1 homolog (*Saccharomyces cerevisiae*); GFP, green fluorescent protein.

Expression levels of NOB1p in several osteosarcoma cell lines were assessed using western blot analysis. NOB1p was moderately expressed in Saos-2 and U2OS cells (Fig. 1A). A lentivirus-mediated RNAi system was applied to specifically downregulate the expression of *NOB1*. To ensure the lentiviral infection efficiency, the expression of green fluorescent protein was detected using fluorescence microscopy. Fig. 1B and C show that, after 96 h of incubation, infection in Saos-2 and U2OS cells was highly efficient (>80%). Knockdown efficiency was determined using qPCR, which showed that the relative levels of *NOB1* mRNA transcripts were significantly decreased by almost 50% in the Lv-sh*NOB1* group as compared with those in the Lv-shCon and Con groups in the two cell lines (Fig. 1D and E). These results demonstrated that endogenous *NOB1* expression was specifically inhibited by the Lv-sh*NOB1* construct.

### Effect of NOB1 silencing on proliferation in human osteosarcoma cells

**Figure 2:**
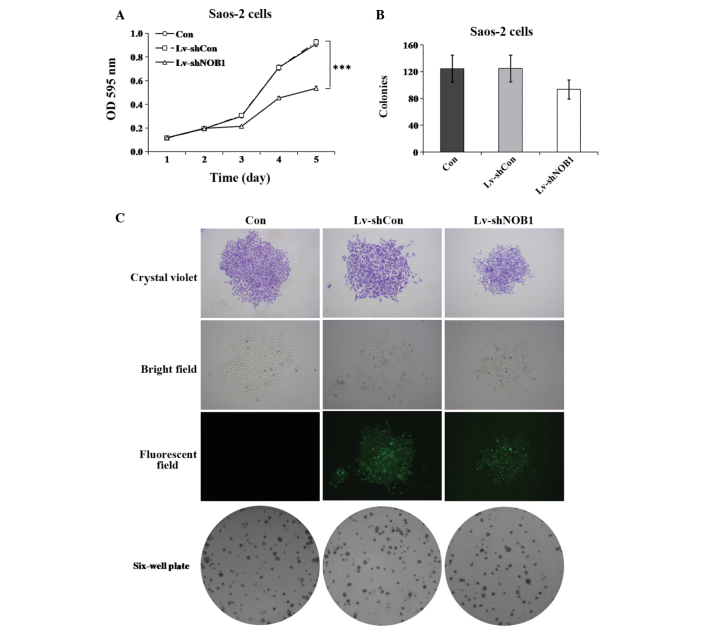
Effect of *NOB1* inhibition on the growth of Saos-2 cells. (A) Cell proliferation assay was performed using methylthiazoletetrazolium staining. The absorbance of the plate was recorded at 595 nm. (B) Saos-2 cells were allowed to grow into natural colonies in a six-well plate. Following staining with Giemsa, the number of colonies was counted in the three groups. (C) Fluorescence and light photomicrographs of Saos-2 cell monoclones in the three groups (magnification, ×40). ^***^P<0.001 compared with Lv-shCon. Con, no lentivirus treatment; Lv-shCon, control lentivirus; Lv-sh*NOB1*, lentivirus containing short hairpin RNA targeting *NOB1; NOB1*, NIN1/RPN12 binding protein 1 homolog (*Saccharomyces cerevisiae*); OD, optical density.

**Figure 3:**
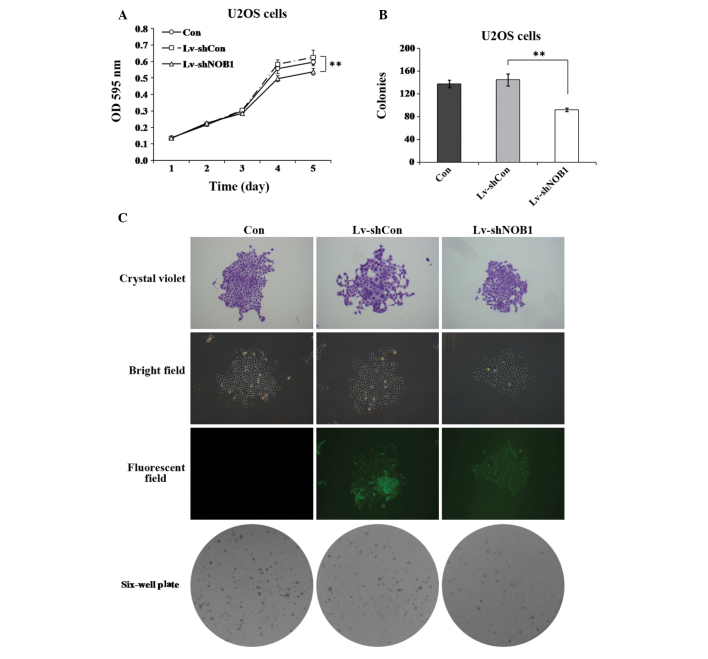
Effect of *NOB1* inhibition on the growth of U2OS cells. (A) Cell proliferation assay was performed using methylthiazoletetrazolium staining. The absorbance of the plate was recorded at 595 nm. (B) U2OS cells were allowed to grow into natural colonies in a six-well plate. Following staining with Giemsa, the number of colonies was counted in the three groups. (C) Fluorescence and light photomicrographs of U2OS cell monoclones in the three groups (magnification, ×40). ^***^P<0.001 compared with Lv-shCon. Con, no lentivirus treatment; Lv-shCon, control lentivirus; Lv-sh*NOB1*, lentivirus containing short hairpin RNA targeting *NOB1; NOB1*, NIN1/RPN12 binding protein 1 homolog (*Saccharomyces cerevisiae*); OD, optical density.

To further investigate the role of *NOB1* in regulating the proliferation of osteosarcoma cells, the MTT assay and colony formation analysis were used. As shown in Fig. 2A, the proliferation of Lv-sh*NOB1*-treated Saos-2 cells at 96 h post-infection was markedly inhibited as compared with that in the Lv-shCon and Con groups (P<0.001). As shown in Fig. 2B and C, the colony formation ability of Saos-2 cells was also slightly reduced by *NOB1* inhibition (Lv-sh*NOB1*, 94±14 colonies), as compared with that of the cells in the Lv-shCon (125±20 colonies; P=0.1) or Con (125±20 colonies; P=0.1) groups. In U2OS cells, similar results were obtained in the two assays (Fig. 3), showing that *NOB1*-knockdown can notably decrease the proliferation of U2OS cells over a short or relatively long period of time. These findings support the theory that *NOB1* has an important role in regulating the growth of osteosarcoma cells.

### Effect of NOB1 silencing on the cell cycle distribution in Saos-2 cells

**Figure 4:**
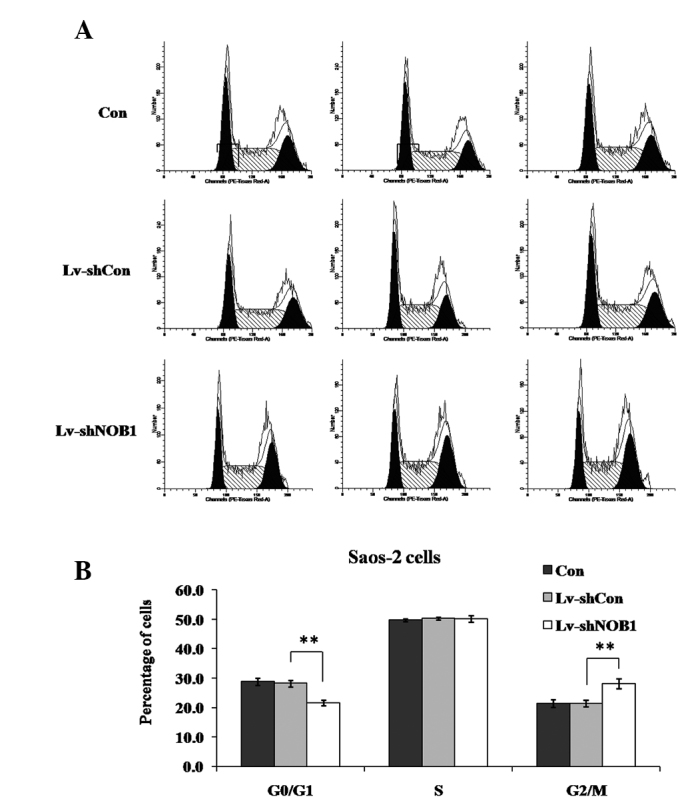
*NOB1* inhibition induces G2/M arrest in Saos-2 cells. (A) Flow cytometry histograms of Saos-2 cells following lentivirus infection in the three groups (Con, Lv-shCon and Lv-sh*NOB1*). (B) Quantification of cell cycle distribution in Saos-2 cells by fluorescence-activated cell sorting. ^**^P<0.01 compared with Lv-shCon. Con, no lentivirus treatment; Lv-shCon, control lentivirus; Lv-sh*NOB1*, lentivirus containing short hairpin RNA targeting *NOB1; NOB1*, NIN1/RPN12 binding protein 1 homolog (*Saccharomyces cerevisiae*).

To examine whether *NOB1*-knockdown suppressed the growth of osteosarcoma cells through direct regulation of the cell cycle, the cell cycle distribution following lentivirus treatment was assessed. The cells were subjected to three different treatments as described previously in the study (Lv-sh*NOB1*, Lv-shCon or Con), and the cell cycle distribution was analyzed using flow cytometry (Fig. 4). In Saos-2 cells, it was observed that, compared with Lv-shCon or Con, Lv-sh*NOB1* significantly decreased the percentage of cells in G0/G1 phase (P<0.01) and increased that in G2/M phase (P<0.01), indicating that *NOB1* suppression induced cell cycle arrest at G2/M phase.

### Effect of NOB1 silencing on cell migration in U2OS cells

**Figure 5:**
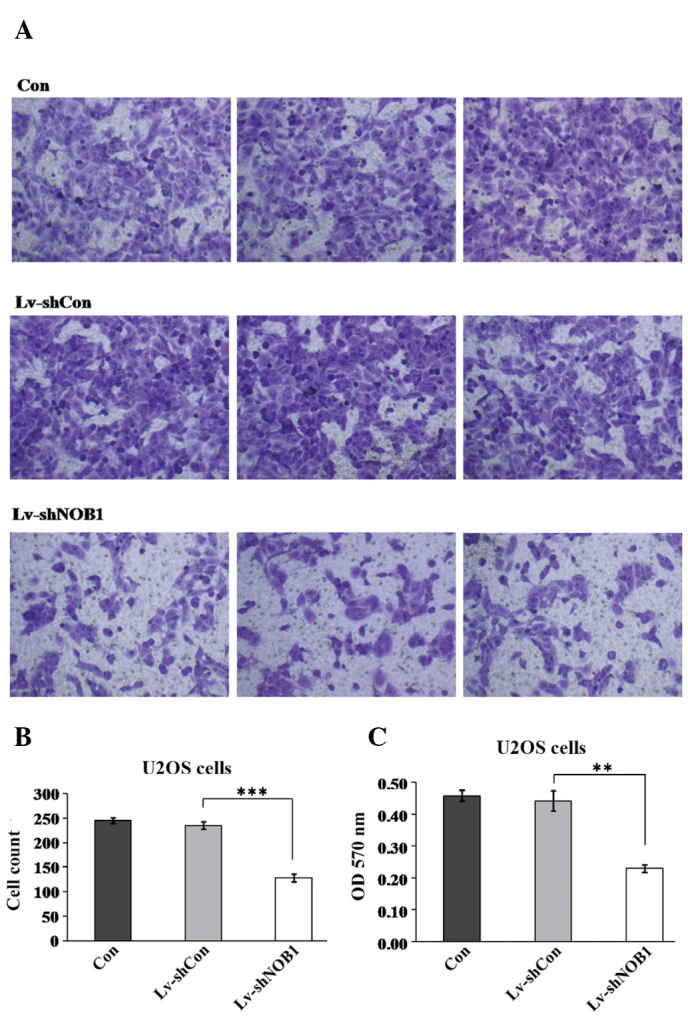
*NOB1* inhibition suppresses U2OS cell migration. U2OS cells were subjected to three treatments (Con, Lv-shCon or Lv-sh*NOB1*) for 72 h. Cells were then seeded onto the upper chamber of the Transwell plate. Migrated cells were (A) stained with crystal violet (magnification, ×100) and (B) counted. (C) Stained cells were dissolved and color intensity was assessed using a spectrophotometer. ^**^P<0.01, ^***^P<0.001 compared with Lv-shCon. Con, no lentivirus treatment; Lv-shCon, control lentivirus; Lv-sh*NOB1*, lentivirus containing short hairpin RNA targeting *NOB1; NOB1*, NIN1/RPN12 binding protein 1 homolog (*Saccharomyces cerevisiae*); OD, optical density.

**Figure 6:**
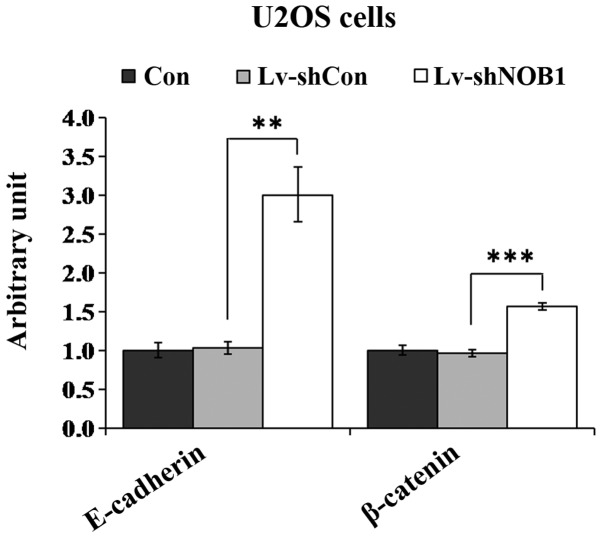
Effect of *NOB1* inhibition on cancer cell regulators. The protein levels of E-cadherin and β-catenin were examined using western blot analysis. ^**^P<0.01, ^***^P<0.001 compared with Lv-shCon. Con, no lentivirus treatment; Lv-shCon, control lentivirus; Lv-sh*NOB1*, lentivirus containing short hairpin RNA targeting *NOB1; NOB1*, NIN1/RPN12 binding protein 1 homolog (*Saccharomyces cerevisiae*).

Cell migration is a critical step that occurs during cancer progression. Therefore, the potential effect of *NOB1* silencing in regulating U2OS cell migration was assessed using the Transwell assay (Fig. 5). Fewer cells in the Lv-sh*NOB1* group (128.2±8.2) migrated into the lower filter as compared with the cells in the Lv-shCon (235.5±8.1) and Con (245.3±5.8) groups. In addition, the crystal violet staining intensity was significantly lower in the Lv-sh*NOB1* group (0.23±0.01) than that in the Lv-shCon (0.44±0.03) and Con (0.46±0.02) groups. This assay indicated that *NOB1*-knockdown strongly suppressed the migration of U2OS cells. Additionally, in order to explore the underlying molecular mechanism of Lv-sh*NOB1* suppressing the proliferation and migration of osteosarcoma cells, the expression of several molecules, including fibronectin, vimentin, N-cadherin, E-cadherin and β-catenin was detected (data not shown). Downregulation of *NOB1* was associated with the increase expression of two regulators, E-cadherin and β-catenin, as compared with expression in the Con or Lv-con groups (Fig. 6). Thus, it is likely that E-cadherin and β-catenin are involved in Lv-sh*NOB1*-mediated growth inhibition in human osteosarcoma cells.

## Discussion

*NOB1* encodes a nuclear protein that regulates the maturation of the 20S proteasome and favors 26S proteasome biogenesis (12). Advances in the investigation of the mechanisms underlying proteasome activity have led to the exploration of proteasome inhibitors as effective drugs against several human cancer and solid tumor types (6–8,10,19). Several studies have demonstrated that bortezomib, alone or in combination with other proteasome inhibitors, is by far the most effective in the induction of apoptosis in osteosarcoma cells (5), indicating that the ubiquitin-proteasome complex (UPP) may have an important role in osteosarcoma. However, the biological function and therapeutic potential of *NOB1*, a key factor in the UPP and proteasome complex, remain to be fully elucidated.

In the present study, a lenti-shRNA system was applied, which effectively inhibited *NOB1* expression at the RNA and protein levels. *NOB1*-knockdown strongly suppressed the growth of osteosarcoma cells and caused G2/M-phase arrest, as confirmed by MTT, colony formation and cell cycle assays. Furthermore, the absence of *NOB1* inhibited osteosarcoma cell motility and migration. It is of note that the expression levels of E-cadherin and β-catenin were significantly increased when *NOB1* was downregulated. These two molecules have been reported to be associated with the metastatic progression of several types of cancer (20–24). Loss of the tumor suppressor genes E-cadherin and β-catenin has been suggested to enable metastasis by disrupting intercellular contacts, which is an early step in metastatic dissemination (21). E-cadherin is also known to associate with a number of proteins, including three catenins (α, β and p120), via its cytoplasmic domain, which links E-cadherin to the actin cytoskeleton (21). Cai *et al* (25) demonstrated that the wingless-type MMTV integration site family (Wnt)/β-catenin pathway is inactivated in osteosarcoma. Moreover, activation of the Wnt/β-catenin pathway inhibits cell proliferation and promotes osteogenic differentiation in osteosarcoma cells. The present results indicate that *NOB1* depletion may inhibit osteosarcoma development by increasing E-cadherin and β-catenin expression.

In conclusion, the present study reported the novel finding that *NOB1* inhibition is able to strongly suppress cell growth and migration of human osteosarcoma cells. Therefore, it is suggested that *NOB1* may be a potential target in developing specific UPP inhibitors for the treatment of osteosarcoma.

## References

[1] Glickman MH, Ciechanover A (2002). The ubiquitin-proteasome proteolytic pathway: destruction for the sake of construction. Physiol Rev.

[2] Ferrell K, Wilkinson CR, Dubiel W, Gordon C (2000). Regulatory subunit interactions of the 26S proteasome, a complex problem. Trends Biochem Sci.

[3] Frezza M, Schmit S, Dou QP (2011). Targeting the ubiquitin-proteasome pathway: an emerging concept in cancer therapy. Curr Top Med Chem.

[4] Yerlikaya A, Yöntem M (2013). The significance of ubiquitin proteasome pathway in cancer development. Recent Pat Anticancer Drug Discov.

[5] Pandit B, Gartel AL (2011). Thiazole antibiotic thiostrepton synergize with bortezomib to induce apoptosis in cancer cells. PLoS One.

[6] Piperdi B, Ling YH, Liebes L, Muggia F, Perez-Soler R (2011). Bortezomib: understanding the mechanism of action. Mol Cancer Ther.

[7] Dick LR, Fleming PE (2010). Building on bortezomib: second-generation proteasome inhibitors as anti-cancer therapy. Drug Discov Today.

[8] Shapovalov Y, Benavidez D, Zuch D, Eliseev RA (2010). Proteasome inhibition with bortezomib suppresses growth and induces apoptosis in osteosarcoma. Int J Cancer.

[9] Pandit B, Bhat UG, Gartel AL (2011). Proteasome inhibitory activity of thiazole antibiotics. Cancer Biol Ther.

[10] Bhat UG, Zipfel PA, Tyler DS, Gartel AL (2008). Novel anticancer compounds induce apoptosis in melanoma cells. Cell Cycle.

[11] Tone Y, Tanahashi N, Tanaka K, Fujimuro M, Yokosawa H, Toh-e A (2000). Nob1p, a new essential protein, associates with the 26S proteasome of growing *Saccharomyces cerevisiae* cells. Gene.

[12] Tone Y, Toh-E A (2002). Nob1p is required for biogenesis of the 26S proteasome and degraded upon its maturation in *Saccharomyces cerevisiae*. Genes Dev.

[13] Oehler VG, Yeung KY, Choi YE, Bumgarner RE, Raftery AE, Radich JP (2009). The derivation of diagnostic markers of chronic myeloid leukemia progression from microarray data. Blood.

[14] Lu Z, Guo Q, Shi A, Xie F, Lu Q (2012). Downregulation of NIN/RPN12 binding protein inhibit the growth of human hepatocellular carcinoma cells. Mol Biol Rep.

[15] Lin Y, Peng S, Yu H, Teng H, Cui M (2012). RNAi-mediated downregulation of Nob1 suppresses the growth and colony-formation ability of human ovarian cancer cells. Med Oncol.

[16] Sakoda T, Kasahara N, Hamamori Y, Kedes L (1999). A high-titer lentiviral production system mediates efficient transduction of differentiated cells including beating cardiac myocytes. J Mol Cell Cardiol.

[17] Soneoka Y, Cannon PM, Ramsdale EE (1995). A transient three-plasmid expression system for the production of high titer retroviral vectors. Nucleic Acids Res.

[18] Pfaffl MW, Horgan GW, Dempfle L (2002). Relative expression software tool (REST) for group-wise comparison and statistical analysis of relative expression results in real-time PCR. Nucleic Acids Res.

[19] Adams BK, Ferstl EM, Davis MC (2004). Synthesis and biological evaluation of novel curcumin analogs as anti-cancer and anti-angiogenesis agents. Bioorg Med Chem.

[20] Lee SJ, Choi SY, Kim WJ (2013). Combined aberrant expression of E-cadherin and S100A4, but not β-catenin is associated with disease-free survival and overall survival in colorectal cancer patients. Diagn Pathol.

[21] Onder TT, Gupta PB, Mani SA, Yang J, Lander ES, Weinberg RA (2008). Loss of E-cadherin promotes metastasis via multiple downstream transcriptional pathways. Cancer Res.

[22] Perl AK, Wilgenbus P, Dahl U, Semb H, Christofori G (1998). A causal role for E-cadherin in the transition from adenoma to carcinoma. Nature.

[23] Derksen PW, Liu X, Saridin F (2006). Somatic inactivation of E-cadherin and p53 in mice leads to metastatic lobular mammary carcinoma through induction of anoikis resistance and angiogenesis. Cancer Cell.

[24] Debald M, Kaiser C, Abramian A (2013). Evaluation of E-cadherin, Ki-67 and lymphatic vessel invasion in abdominal metastases of human breast cancer. Anticancer Res.

[25] Cai Y, Mohseny AB, Karperien M, Hogendoorn PC, Zhou G, Cleton-Jansen AM (2010). Inactive Wnt/beta-catenin pathway in conventional high-grade osteosarcoma. J Pathol.

