# Gene silencing of NOB1 by lentivirus suppresses growth and migration of human osteosarcoma cells

**DOI:** 10.3892/mmr.2022.12909

**Published:** 2022-12-06

**Authors:** Bingpeng Chen, Jingjing Liu, Dankai Wu, Yanguo Qin, Chuangang Peng, Chen Li, Jincheng Wang

Mol Med Rep 9: 2173–2179, 2014; DOI: 10.3892/mmr.2014.2119

Subsequently to the publication of this paper, an interested reader drew to the authors’ attention that, on p. 2174 in the Materials and methods section (subsection “*Lentivirus production and lentiviral transduction*”), the sequence presented for the shRNA targeting the gene *NOB1* appeared to conform with the sequence that would have been predicted to target *PNO1*, according to a BLASTN search. The authors have checked their original paper, and realize that the sequence of this shRNA was written incorrectly in the paper; the sequence for the shRNA targeting the gene *NOB1* should have been written as: GCTTGCACTCACATACCAGTTCTCGAGAACTGGTATGTGAGTGCAAGC.

Furthermore, the published version of Fig. 5A on p. 2178 contained a pair of overlapping panels, such that data were apparently derived from the same original source even though they were intended to show the results from differently performed experiments. After having re-examined their original data, the authors have realized that a pair of data panels were inadvertently incorporated into this figure incorrectly; specifically, the centre panel of the Lv-shCon group and the right-hand panel of the Lv-shNOB1 group.

The revised version of Fig. 5, showing the correct images for the abovementioned pair of data panels in Fig. 5A, is shown opposite. Note that these errors did not significantly affect either the results or the conclusions reported in this paper, and all the authors agree to this corrigendum. Furthermore, the authors thank the Editor of *Molecular Medicine Reports* for allowing them the opportunity to publish this corrigendum, and apologize to the readership for any inconvenience caused.

## Figures and Tables

**Figure 5.**
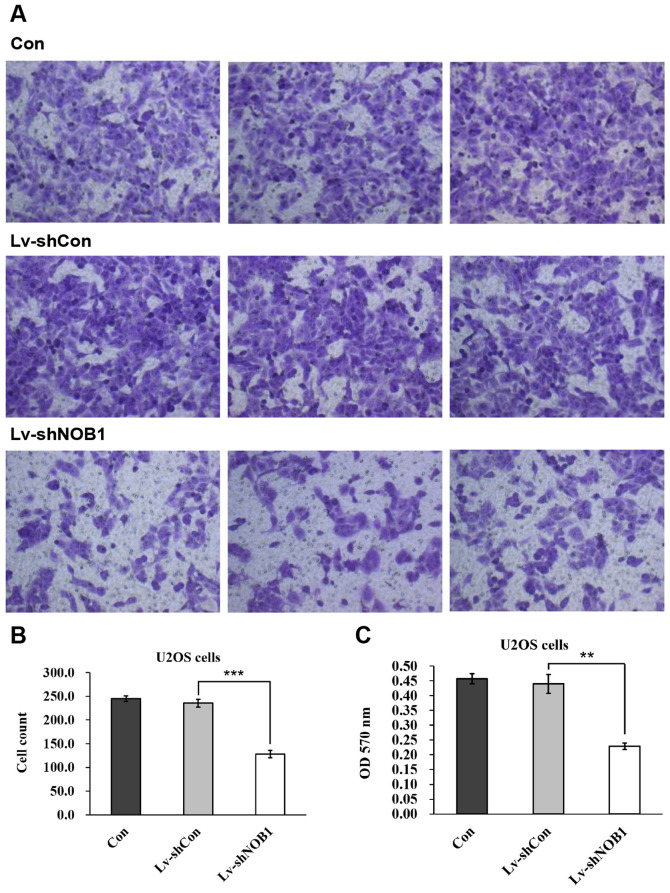
NOB1 inhibition suppresses U2OS cell migration. U2OS cells were subjected to three treatments (Con, Lv-shCon or Lv-shNOB1) for 72 h. Cells were then seeded onto the upper chamber of the Transwell plate. Migrated cells were (A) stained with crystal violet (magnification, ×100) and (B) counted. (C) Stained cells were dissolved and color intensity was assessed using a spectrophotometer. **P<0.01, ***P<0.001 compared with Lv-shCon. Con, no lentivirus treatment; Lv-shCon, control lentivirus; Lv-shNOB1, len-tivirus containing short hairpin RNA targeting NOB1; NOB1, NIN1/RPN12 binding protein 1 homolog (*Saccharomyces cerevisiae*); OD, optical density.

